# Emerging roles of lncRNA in Nasopharyngeal Carcinoma and therapeutic opportunities

**DOI:** 10.7150/ijbs.70292

**Published:** 2022-03-28

**Authors:** Haihua Wang, Weiyuan Wang, Songqing Fan

**Affiliations:** 1 Department of Pathology, The Second Xiangya Hospital, Central South University, Changsha, Hunan, 410011, China.; 2 Department of Pathology, The Xiangya Hospital, Central South University, Changsha, Hunan, 410008, China.

**Keywords:** Long-non-coding RNAs, Nasopharyngeal Carcinoma, emerging roles, therapeutic opportunities

## Abstract

Nasopharyngeal carcinoma (NPC) is a kind of malignant tumor with ethnic and geographical distribution characteristics. However, the molecular mechanisms of NPC are still unclear. Long non encoding RNAs (lncRNAs) are becoming important regulators in gene expression networks, including post transcriptional and post translational regulation of protein, protein complex organization, signal transduction and recombination among cells, which are involved in cancer recognition. Recent evidence shows that lncRNAs play important roles in the occurrence and development of NPC. Therefore, in-depth understanding of abnormal lncRNAs will provide new understanding of the pathogenesis in NPC, and provide new tools for the early diagnosis and treatment of NPC. This article reviews the abnormal lncRNAs in NPC cells and the roles of lncRNAs in the tumorigenesis of NPC. In addition, we also discuss the diagnostic and therapeutic potential of lncRNAs in NPC.

## Introduction

Nasopharyngeal carcinoma (NPC) is a unique malignant tumor of the head and neck, which originates from the epithelial of the nasopharyngeal mucosa. Nevertheless, the global incidence rate is uneven. Over 70% of new cases occur in East Asia and Southeast Asia^1^. The exact etiology of NPC remains unclear, but risky factors recognized include Epstein Barr Virus (EBV) infection, genetic predispositions, dietary habits and environmental factors^2^. Most of them have stepped into advanced stage when they are diagnosed for the first time. So far, the intensity modulated radiotherapy (IMRT) remains the main treatment for non-metastatic nasopharyngeal carcinoma^3^, ^4^. The NCCN guidelines recommend concurrent chemoradiotherapy combined with adjuvant chemotherapy, or induction chemotherapy combined with radiotherapy / chemoradiotherapy for locoregionally advanced NPC (T1, N1-3; T2-T4, any N)^5^. Although the survival rate of early NPC after radiotherapy and chemotherapy is high, the survival rate of patients with metastatic or recurrent NPC (IVB stage) is still low. Besides, radiotherapy or Chemoradiotherapy will cause acute and residual toxicity inevitably, such as dry mouth, sensorineural hearing loss, radiation osteonecrosis and a series of complications, leading to the decline of the life quality of patients^1^. In recent years, immune checkpoint therapy has made great progress, which is a promising method to improve clinical outcomes. Studies have shown that these patients benefit from a heterogeneous population. Thus, looking for more targeted and individual differences in treatment still has important clinical application prospects.

As we all know, RNA plays a vital role in the organization and regulation of genome through the activity of a large number of protein-coding or non-protein coding RNAs (ncRNAs). On the basis of the Encyclopedia of DNA Elements (ENCODE) project, the transcripts cover 62-75% of our genome, most of which are non-coding RNA^6^. Long non-coding RNA (lncRNA) usually defined as non-protein coding RNA molecules longer than 200 nucleotides, is once considered as transcription "noise". However, more and more studies have shown that lncRNAs play different roles in regulating gene transcription, post transcription, post translation and epigenetic modification by interacting with DNA, RNA, protein molecules and / or their combinations during gene expression, and the abnormal expression or deficiency of them closely related to tumor occurrence and development^7^-^10^. In addition, evidence shows that lncRNAs can act as either tumor suppressor genes or oncogenes in tumor progression by regulating tumor suppressor or oncogenic mRNAs respectively through numerous mechanisms, including the epigenetic, transcriptional, post transcriptional regulation of relative genes, cell cycle distribution, cell differentiation, epigenetic modification control and so on^11^-^14^. The RNA-based therapeutics are also gradually playing roles in a variety of cancers^15^. In recent years, the occurrence and mechanism of lnRNAs in NPC have been widely studied. Many lncRNAs are maladjusted in NPC, and play important roles in the occurrence, development and even treatments of NPC. In this review, we will describe the biological role and mechanism of lncRNAs in NPC, aiming to clarify the latest research on the potential use of lncRNAs to be diagnostic / prognostic markers and therapeutic targets.

### Aberrant levels of lncRNAs in NPC

**Figure 1:**
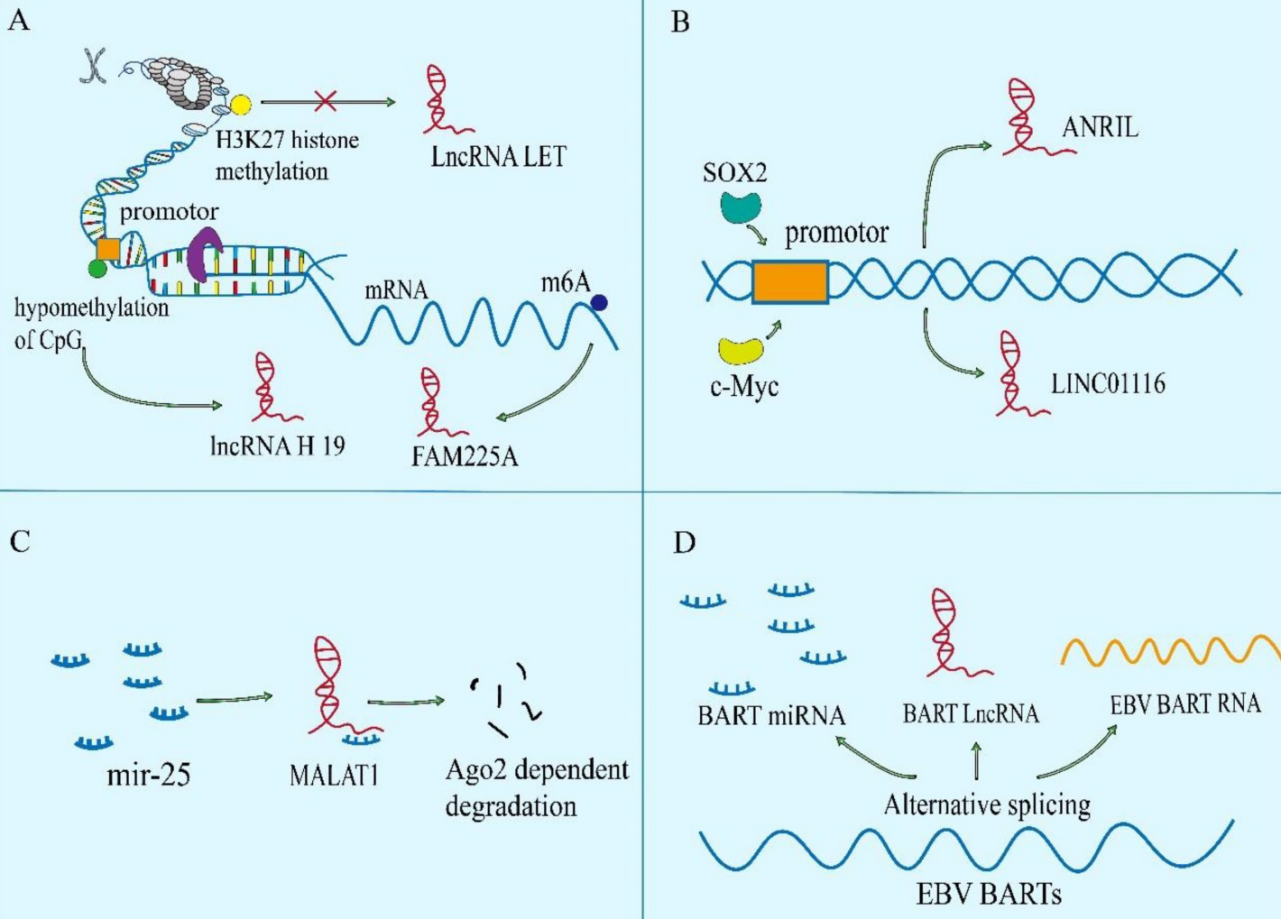
** The regulation of the levels of lncRNAs in NPC. A.** The epigenetic regulation of lncRNAs: LET can be transcriptional repressed by H3K27 histone methylation on the LET promoter. Modification of m6A in FAM225A leads to the improvement of RNA stability of it. The CpG dinucleotides within the H19 promoter region correlates with well‐differentiated NPC. **B.** The transcriptional activation of lncRNAs by some oncogenic transcription factors (e.g., c-Myc and SOX2). **C.** The binding of microRNAs to lncRNAs. **D.** The special processing methods that endow lncRNAs with carcinogenic function.

A number of genome sequencing show that some lncRNAs are dysregulated during the development of NPC. Abnormal lncRNAs biogenesis is related to the pathogenesis of NPC. The different lncRNA profiles of NPC are shown in Table 1. The imbalance of lncRNAs in the occurrence and development of NPC mainly include the epigenetic regulation, the transcriptional activation by some oncogenic transcription factors, the binding of microRNAs to lncRNAs, and the special processing methods that endow lncRNAs with carcinogenic function (Figure 1).

### Epigenetic modification

Epigenetic regulation is the mechanism by which gene function is selectively activated or inactivated in the cells^16^, ^17^. Similar to mRNAs, lncRNAs can also be controlled by epigenetic regulation^18^. In NPC, abnormal chromatin marks at lncRNA genes can be found a trace (Figure 1A). The lncRNA-LET is transcriptionally repressed by H3K27 histone methylation on the LET promoter mediated by enhancer of zeste homolog 2 (EZH2) 19. And EBV-encoded latent membrane protein 1 (LMP1) up-regulates the expression of EZH2 20. In addition, m6A modification in lncRNA is extremely widespread, which has been shown to participate in the occurrence and development of various cancers. In NPC, it is also found that m6A is enriched on lncRNA-FAM225A. Modification of m6A in FAM225A leads to the improvement of RNA stability, which may partially account for upregulation of FAM225A in NPC. Mechanistically, up-regulated FAM225A functions as a competing endogenous RNA (ceRNA) for sponging miR-590-3p and miR-1275, leading to the upregulation of their target integrin subunit β 3 (ITGB3), and the activation of FAK/PI3K/Akt signaling to promote NPC cell proliferation and invasion^21^.

### Transcriptional activation

Recently, it has been reported that many transcription factors may be involved in the regulation of transcription of lncRNAs in NPC (Figure 1B), involving to c-Myc, SOX2 and androgen receptor (AR)^22^-^24^. For example, increased SOX2-binding activity on the ANRIL promoter induces the transcription of ANRIL^22^. Furthermore, AR is a transcription factor that acts as a steroid hormone and has been found to mediate the increased transcription of linc01503^23^. As well as we know, the c-Myc proto-oncogene is a significant member of the Myc/Mad/Max transcription factor network, which regulates nearly 15% of genes in the human genome^25^, ^26^, thus participating in the various pathological of tumor. It is noteworthy that c-Myc has two binding sites in the region of lncRNA-AFAP1-AS1, which can control the promoter of lncRNA-AFAP1-AS1^24^. MYC also serves as the transcriptional activator of LINC01116 in NPC cells^27^.

### miRNA sponging

At present, the ceRNA mechanism has been studied more, but some studies have found that miRNAs can sponge lncRNAs, thereby regulating the level of lncRNAs itself by post transcriptional regulation^28^, ^29^. For instance (Figure 1C), elevated miR-25 in NPC cells can bind to the metastasis associated lung adenocarcinoma transcript 1 (MALAT1) for directly silencing it in an Argonaute2 (Ago2)-dependent degradation, thereby inhibiting tumor progression^28^. However, for the present, the relationship between miRNAs and lncRNAs is mainly focused on the mechanism of ceRNA, but miRNAs are another way to decrypt lncRNAs.

### RNA processing

Like coding proteins, noncoding RNAs are cut and transcribed from the genome. Different splicing methods produce different functional molecules, which are partly involved in the occurrence of NPC (Figure 1D). For example, EBV BARTs comprise two groups of non-coding RNAs. One is a group of miRNAs which are produced from introns prior to splicing. And the other is a complex family of alternatively spliced polyadenylated RNAs^30^. Among the multiple splicing forms of BART RNA, lncRNA BART can regulate host gene expression, maintain EBV latency associated environment and drive carcinogenesis in NPC^31^.

### Functional mechanisms of lncRNAs in NPC

**Figure 2:**
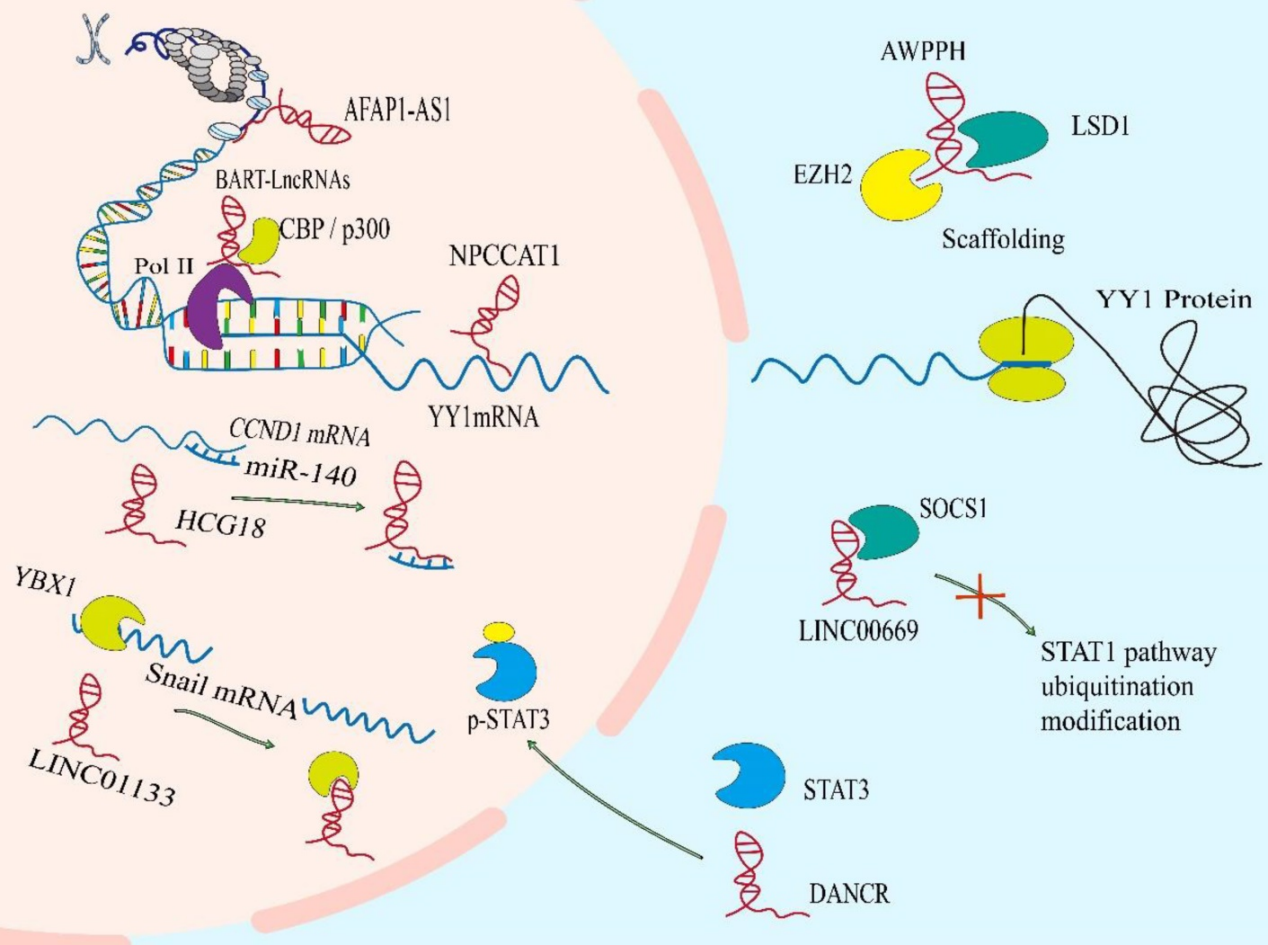
** Functional mechanisms of lncRNAs in NPC.** The lncRNAs can reshape chromatin structure and epigenetic modification. They act as molecular sponges to interact with mRNAs or miRNAs to regulate the stability and translation of mRNAs or the binding of miRNAs to their own targets. LncRNAs can regulate the location or stability of protein via binding to it, and can also regulate the formation or dissociation of protein complex, to adjust the function of protein.

LncRNA is a class of multifunctional molecules. The versatility of lncRNA stems from several unique physical and chemical properties^32^. Firstly, and perhaps most obvious, it is the ability to pair with other nucleic acid bases. The lncRNA is particularly good at recognizing DNA and RNA targets through simple one-to-one base pairing interactions. By binding to DNA or RNA, lncRNAs can reshape chromatin structure and epigenetic modification. Furthermore, lncRNAs act as molecular sponges to interact with mRNAs or miRNAs to regulate the stability and translation of mRNAs or the binding of miRNAs to their own targets. Secondly, lncRNA molecules can be folded into three-dimensional structures of complex to provide a complex recognition surface, thus regulating the location or stability of protein via binding to it, and the formation or dissociation of protein complex, to adjust the function of it (Figure 2)^33^.

### DNA binding and chromatin remodeling

Nuclear lncRNAs have been widely observed to be associated with DNA by RNA-DNA binding. This transcriptional regulation through lncRNA-DNA interaction usually changes chromatin structure through forming RNA-DNA heterozygotes (also known as R loops)^34^, which may provide signals to recruit biomolecules (for instance, epigenetic modifiers) to regulate gene expression, or to spatially aggregate promoters, enhancers or inhibitors to regulate transcriptional activity.^35^, ^36^ The lncRNA BamHI-A rightwards transcripts (BARTs) localize within the nucleus of EBV-infected cells, and appear to stall RNA polymerase ll at the promoter region of IFNB1 and CXCL to inhibit transcription. It suggests that lncRNA BART mediates epigenetic regulation of gene expression through interaction with the chromatin remodeling machinery^31^. The lncRNAs can also regulate the acetylation level of downstream gene promoters. The AFAP1-AS1 binds to KAT2B and promotes acetyltransferase activation at two residues (E570/D610). Then KAT2B further promotes H3K14 acetylation and protein binding to the bromo domain of TIF1α. Consequently, TIF1α acts as a nuclear transcriptional coactivator of RBM3 transcription, leading to stabilization of mRNA of YAP and enhancing NPC tumorigenicity^37^. Some lncRNAs act as cores or scaffolds. The lncRNA ANCR may act as a scaffold to stable the interaction of EZH2 and other proteins within or without PRC2 complex, which is vital in the regulation of target genes of EZH2^38^. The lncRNA PVT1 also serves as a scaffold for the chromatin modification factor KAT2A to mediate histone 3 lysine 9 acetylation (H3K9), thereby recruiting the nuclear receptor binding protein TIF1β to activate NF90 transcription, increasing HIF-1α stability and promoting a malignant phenotype of NPC cells^39^. The hypo‐ and hypermethylation of CpG dinucleotides have also been demonstrated to be an important epigenetic event in the regulation of gene transcription by modifying the structure of chromatin^40^. And hypomethylation of the CpG dinucleotides within the lncRNA H19 promoter region correlates with well‐differentiated NPC^41^.

### Sponging mRNAs and miRNAs

It is noteworthy that specific endogenous lncRNAs containing miRNA binding sites can function as sponge for specific miRNAs and interfere with their functions, thereby regulating gene expression. Significantly, lncRNAs may interact with a tremendous amount of miRNAs and weaken their ability to bind to mRNAs^42^. Such as, SNHG1, CYTOR, MALAT1, nuclear paraspeckle assembly transcript 1(NEAT1) and so on, they all have ability to sponge with miR-145-5p, miR‐613, miR-124 or miR-204, respectively^43^-^46^. Mechanistically, a novel and extensive interaction network involving ceRNAs, in which lncRNAs regulate protein-coding mRNA molecules by competitively binding their target sites to miRNAs^47^. The lncRNA-miRNA axis is involved in the regulation of various cell signaling pathways, like PI3K / Akt / mTOR, Wnt / β-Catenin, Hedgehog, NF-κB, NOTCH3, Ras-MAPK, ubiquitin-specific protease 39 and so on, which are closely related to the occurrence and development of NPC^21^, ^48^-^54^. A case in point is that lncRNA HCG18 modulates Wnt/β-catenin and Hedgehog pathway by directly binding to miR-140 and effectively act as a ceRNA for miR-140 to increase the expression of cyclin D1, which contributes to NPC progression^55^.The lncRNA ANRIL induces proliferation, inhibits apoptosis, and reduces radiosensitivity in NPC cells through sponging miR-125a^56^. In NPC, there are still many studies on ceRNA, and new lncRNAs have been reported continuously.

### Protein interaction and regulation

In addition to the regulation of gene expression events mediated by the effect of lncRNA on mRNA, the ability of lncRNAs to bind and regulate protein activity goes beyond the factors involved in transcription. These connections mainly involve in the association of lncRNAs with proteins orchestrates protein localization or stability, as well as protein complex assembly or sequestration of proteins from their own binding partners, thereby executing their biological function.

#### Protein translation

Although lncRNAs do not have open reading circles for protein translation, ribosome profiling has identified ribosome-associated lncRNAs^57^. Meanwhile, many studies have showed that the distribution of lncRNAs in cytoplasm can regulate the expression of proteins via directly interacting with them^34^. The LINC01116 in the cytoplasm can enhance MYC protein translation as antisense transcripts facilitating the translation of selected mRNA in a cap‐dependent manner^27^. In the same way, NPC copy number amplified transcript-1 directly binds to the 5'UTR of the mRNA of YY1, thereby inducing translation of it, which promotes NPC cell proliferation and migration^58^.

#### Protein localization or stability

It has been reported that the subcellular localization of many tumor-associated proteins is regulated by lncRNAs. Like the lncRNA THOR which can enhance the transcriptional activity of YAP by directly binding to YAP to inhibit translocation of it from nucleus to cytoplasm^59^. The lncRNAs also control protein localization indirectly. The Phosphorylated STAT3 is the active form of STAT3. Overexpressed p-STAT3 is confirmed in more than 70% of NPC patients^60^. The LINC00669 can competitively bind to SOCS1, the key suppressor of JAK/STAT signaling pathway, and insulate it from imposing ubiquitination modification on the component of JAK/STAT pathway, which leads to the augment of both total and phosphorylation of STAT3, and transfers it from cytosolic into nucleus for initiating the transcription of genes concerned about proliferation and invasion in NPC^61^. Meanwhile, lncRNA DANCR seems to function as a molecular scaffold to promote STAT3 activation by facilitating JAK1 to phosphorylate STAT3 and regulate the location of p-STAT3^62^.

Besides, lncRNAs mediate oncoprotein stabilization and tumor suppressor genes lead to the progression or inhibition of NPC, respectively. For instance, lncRNA AWPPH can stabilize LSD1 via interacting with a common RNA binding protein IGF2BP1, which boosts LSD1 expression in cytoplasm of NPC cells, so as to drive NPC process^63^.

#### Protein complex assembly

LncRNAs can also bind to proteins or protein complexes performing explicit biological functions, such as epigenetic regulation^64^. The Adenylate and uridylate (AU)-rich elements (AREs) in the 3´ regions of DANCR has strong binding ability with NF90/NF45 complex, and can interact directly with NF90/NF45 to stabilize HIF-1 α mRNA promoting NPC metastasis^65^. In addition, DANCR complexes with RNA binding protein 3 (RBM3) protein to stabilize SOX2 mRNA, leading to NPC cell proliferation^66^. These lncRNAs participate in the assembly of protein complex, which are more conducive to the implementation of function.

#### Sequestration of proteins from their own binding partners

On the contrary, some lncRNAs act as competitive binding partners to isolate proteins from their original substrates, leading to the dissociation of protein complexes. A well-known RNA and DNA binding protein YBX1 activates *Snail* translation directly and induces epithelial-mesenchymal transition^67^. In NPC, EPB41L4A-AS2^68^ and LINC01133 69 can bind to YBX1 to sequestrate the mRNA of *Snail* from YBX1, thereby suppressing the expression of Snail and inhibiting the epithelial mesenchymal transition (EMT) process of NPC cells. And LINC00669 competitively binds to SOCS1, the key suppressor of JAK/STAT signaling pathway, to insulate it from imposing ubiquitination modification on the pathway component of STAT1, which leads to abnormal stabilization and activation of it to promote NPC cell proliferation and invasion^61^.

### Significance of lncRNAs in NPC hallmarks

**Figure 3:**
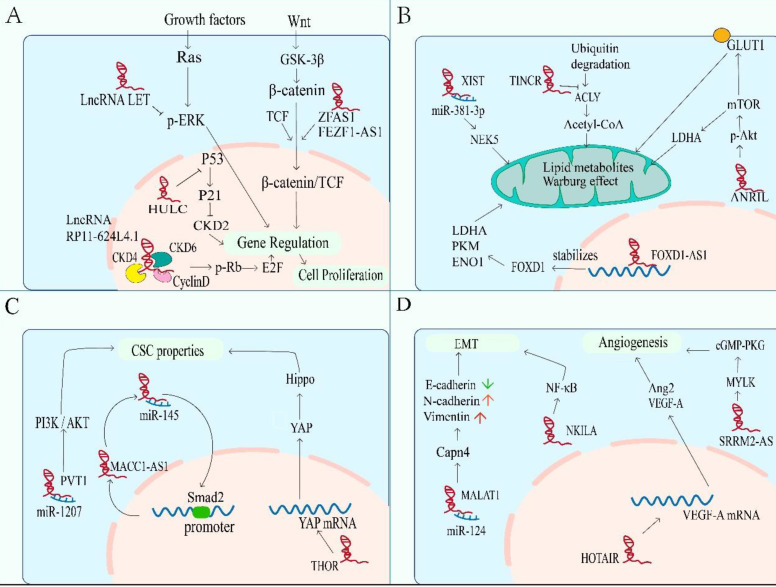
** Significance of lncRNAs in NPC hallmarks.** Nasopharyngeal carcinoma related lncRNAs can promote or inhibit carcinogenic phenotypes through distinct mechanisms. These hallmarks include: **A.** The abnormal regulation of lncRNA leads to sustaining proliferative signaling in NPC. **B.** LncRNAs are involved in dysregulated energetics caused by metabolic reprogramming. **C.** LncRNAs participate in hyperactivation of CSC-related signaling, leading to acquiring CSC properties of NPC cells. **D.** Enhanced metastasis induced by the overexpression of mesenchymal markers and the formation of additional vessels.

Different cancers have similar characteristics, such as continuous proliferation, resistance to cell death and enhancement of invasion. These characteristics are closely related to cancer behaviors^70^. NPC related lncRNAs can promote or inhibit these carcinogenic phenotypes, thus providing a reasonable treatment strategy for NPC (Figure 3).

### Sustaining proliferative signaling

One of the most remarkable characteristics of cancer cells is the growth signal of self-sufficiency, even without external stimulation. Normal cells carefully control the production of growth promoting or inhibiting factors to ensure strict control of cell number, tissue structure and function. On the contrary, tumor cells show uncontrolled growth response, which makes them more or less independent of proliferation signals, leading to unlimited expansion. In order to achieve this independence, tumor cells have the acquired ability to maintain proliferation signal transduction in a variety of ways, including the participation of lncRNAs (Figure 3A). Firstly, Cell cycle progression is essential for cell proliferation. The abnormal regulation of cyclin D1, CDK4 and CDK6 is positively correlated with the proliferation of NPC cells. The CDK4 has been identified as a major carcinogenic driver in cell cycle components, belonging to the cyclin dependent kinase family. As a G1 serine / threonine kinase, CDK4 is also an important protein for normal cell proliferation^71^. The CDK4 / cyclin D1 complex phosphorylates and inhibits the tumor suppressor protein retinoblastoma (RB), resulting in the release of E2F transcription factor, which regulates the gene expression required for S-phase entry and progression^72^. The lncRNAs can also participate in the evolution of the cell cycle regulated by CDK4/6 or Cyclin D1 in NPC. For example, lncRNA RP11-624L4.1 acting as a scaffold for CDK4, CDK6, and Cyclin D1 to form a protein complex, which promotes NPC proliferation in the CDK4/6-Cyclin D1-Rb-E2F1 pathway^73^. Secondly, the ERK1 / 2 pathway is also an important signal pathway of proliferation, which can regulate the transition of G1- to S- phase^74^. In NPC, LncRNA-LET suppresses cell growth and cell cycle by inhibiting the phosphorylation of kinase activity of ERK1/2. LET can also up-regulate cell cycle related genes, including *CDKN1A*, *CDKN1B* and *CDKN2D*, and induce cell cycle arrest in G0 / G1 phase^75^. Thirdly, β-catenin is also a cell cycle regulator. When the Wnt signaling is abnormally activated, β-catenin protein cannot be degraded by GSK-3β, resulting in the accumulation of β-catenin in the cytoplasm or the translocation of it into the nucleus, and initiating the transcriptional regulation of downstream target genes^76^. LncRNA ZFAS1 is overexpressed in NPC to promote the occurrence of NPC by activating the Wnt/β-catenin pathway^77^. Besides, lncRNA FEZF1-AS1 also can enhance β-catenin entry of nucleus and result in a series of checkpoint proteins expressing (including CDK4 / 6, c-MYC and CyclinD1) and G1 / S phase transiting and cell growing^78^. Lastly, as well as tumor suppressor protein p53, which is a key protein in cell cycle checkpoint DNA damage detection (by inducing p21 transcription to stop cell cycle in G1 phase). The upregulated lncRNA highly upregulated in liver cancer (HULC) in NPC can inhibit the activation of p53 and down regulate the level of p21 gene, result in unlimited proliferation of tumor cells and tumor progression^79^.

### Dysregulating cellular energetics

Metabolic reprogramming is a sign of cancers. The most obvious is that glycolysis is the main way of energy supply. Although oxidative phosphorylation can produce more ATP, cancer cells still tend to generate energy from glycolysis, which is due to proliferating cells need not only ATP, but also nucleic acids, fatty acids, proteins and membrane phospholipids. Glycolysis can provide the substrates and intermediates needed for the synthesis of these biomacromolecules to meet the needs of rapid DNA replication of tumors. This phenomenon is known as “Aerobic glycolysis "or " Warburg effect”^80^-^83^. An increasing evidence indicates that lncRNAs can regulate glucose metabolism in cancer cells in different ways (Figure 3B), such as directly regulating glycolytic enzymes (e.g., pyruvate carboxylase, fructose-2, 6-bisphosphatase, 6-phosphogluconate, phosphoenolpyruvate carboxy kinase and glucose transporter GLUT), or through indirect regulation of signal transduction pathways (e.g., Wnt/Snail, JAK/STAT, p53 pathways, HIF-1α, PI3K/Akt/mTOR and lkb1-ampk pathways)^83^, ^84^. Glucose transporter is composed of two membrane related carrier proteins, Na + SGLTs and GLUTs^85^. Some lncRNAs regulate the glycolysis process by combining with GLUT. One example is lncRNA ANRIL promotes Akt phosphorylation and activates mTOR signaling pathway, thereby elevating the expression of GLUT1 and LDHA (key enzyme to catalyze the conversion of pyruvate to lactate in the last step of aerobic glycolysis^86^, so as to increase glucose uptake, and rapidly generate ATP for proliferation of NPC cells ultimately^87^. Another example is lncRNA FOXD1-AS1, which promotes glycolysis by maintaining the expression of FoxD1, which is related to lactate dehydrogenase A (LDHA), pyruvate kinase M2 (PKM) and enolase 1 (ENO1) genes^88^.

Compared with nonmalignant cells, cancer cells not only show dysregulation of carbohydrates, but also have alterations related to lipid metabolism^89^. In fact, increased lipogenesis is considered to be a marker of many invasive cancers^90^, with de novo fatty acid (FA) synthesis, for supporting membrane body biosynthesis and the energy requirement of proliferation, and for helping synthesize macromolecules in membranes and deliver lipid signals to fight pressure^90^, ^91^. Acetyl CoA synthesis of lipids is the most common mechanism of lipid supply in tumor cells^92^. In NPC, lncRNA TINCR can protect ATP citrate lyase (ACLY) from ubiquitin degradation to maintain total cellular acetyl-CoA levels. Accumulation of cellular acetyl-CoA promotes de novo lipid biosynthesis and histone H3K27 acetylation, which ultimately regulates the peptidyl arginine deiminase 1 (PADI1)-MAPK-MMP2/9 pathway, and then promoting NPC proliferation, metastasis and cisplatin resistance^93^.

### Acquiring CSC properties

In recent years, cancer stem cells (CSC) have become one of the hot spots in cancer researches, and they are considered as the root of tumor growth, proliferation and recurrence^94^, ^95^. Previous reports indicate that CSCs, similar to embryonic stem cells (ESCs), have the abilities of self-renewal and self-differentiation^96^. These suggest that there may be common signal transduction pathways and molecular markers between CSCs and ESCs. LncRNAs are the necessary regulatory factors for many developmental pathways, including maintaining the pluripotency of stem cells, regulating apoptosis, generating red blood cells, and differentiating horn cells^97^, ^98^. For NPC, several stem cell markers have been identified, such as Oct4, c-Myc, Sox2 and ALDH, which are believed to play a key role in the progression of NPC and chemoresistance^99^. Importantly, multiple signaling are involved in the maintenance of NPC stem cells, such as PI3K/Akt, Wnt-β-catenin, JAK/STAT, and Hippo-YAP pathways^100^. The lncRNAs also participate in the regulation of CSC properties (Figure 3C). The lncRNA PVT1 promotes cancer stem like properties in NPC cells by inhibiting miR-1207 and activating PI3K/Akt signaling pathway^101^. Besides, a novel MACC1-AS1/miR-145/Smad2 negative loop responsible for NPC cell stemness. Chen et al. found that MACC1-AS1 act as a ceRNA to antagonize the activity of miR-145 which can target Smad2. In turn, Smad2 can bind to MACC1-AS1 promoter and increase MACC1-AS1 expression, forming a new MACC1-AS1/miR-145/Smad2 negative loop responsible for NPC cell stemness^102^. Transcriptional factor YAP is one of the executors of Hippo pathway which is a kinase cascade involving mammalian STE20-like protein kinase 1 (MST1) and the large tumor suppressor 1 (LATS1) and LATS2, in which YAP is phosphorylated and inactivated by LATS1/2^103^. Previous study has shown that YAP can confer stemness on tumor cells^104^, and YAP has been considered as a “stemness factor” in several types of stem cells^105^. But the lncRNA THOR can attenuate the cisplatin sensitivity of NPC cells by enhancing cell stemness via promoting YAP transcriptional activity^59^.

### Activating invasion and metastasis

Tumor metastasis leads to the vast majority of cancer-related deaths. In short, EMT and angiogenesis play major roles in tumor metastasis, including NPC. Epithelial-mesenchymal transition is a biological process involving the adhesion between cells, which is related to the migration of cancer cells. During the process, epithelial markers (E-cadherin, ZO-1, etc.) are down regulated, while mesenchymal markers (N-cadherin, vimentin, ZEB1, etc.) are up-regulated^106^, ^107^. LncRNAs also participate in it of NPC (Figure 3C). Such as the lncRNA MALAT1, it can be used as a competitive endogenous RNA (ceRNA) to competitively sponge with miR-124, thus releasing the inhibited target Capn4, which promotes the progress of NPC by reducing the expression of E-cadherin and increasing the expression of N-cadherin and vimentin^45^. As well as the lncRNA NEAT1, which regulates miR-204-mediated ZEB1 to promotes epithelial to mesenchymal transition and radioresistance through miR-204/ZEB1 axis in NPC^46^. In addition to miRNA sponge, lncRNAs can also directly regulate the expression of EMT related molecules, thus affecting the signaling pathway, including NF-κB, PI3K / Akt, MEK / ERK / c-Myc and Rho / RAC signaling pathway^108^-^111^. For example, the expression level of NKILA is enhanced in high metastatic cells than others. In mechanism, NKILA inhibited NF - κ B activation by suppressing IKK induced I κ B phosphorylation in NPC^111^.

Angiogenesis is a key step in tumor development. And new angiogenesis is essential for the production of lethal tumor masses^112^, ^113^. In the development of tumor, pathological angiogenesis is driven by the overexpression of angiogenic factors including lncRNAs, which causes local imbalance between angiogenic and antiangiogenic factors, thus leading to the recruitment of new vascular supply (Figure 3D)^113^, ^114^. In NPC, the HOX transcript antisense RNA (HOTAIR) is found to activate the transcription of VEGF-A and up-regulate the expression of glucose regulated protein 78 (GRP78), then up-regulating the expression of VEGF-A and Ang2 to promote angiogenesis^115^. Therefore, blocking VEGF / VEGFR signal transduction to affect vascular production and destroy nutrition and oxygen supply can provide treatment strategies^114^, ^116^. Another example is serine/arginine repetitive matrix protein 2-alternative splicing (SRRM2-AS), which exerts facilitating effects on angiogenesis in NPC via activating MYLK-mediated cGMP-PKG signaling pathway^117^. And angiostatin (caplostatin and endostatin) has been approved for the treatment of tumor and achieved good results^118^.

### LncRNAs as a modulator of microenvironment associated with NPC

**Figure 4:**
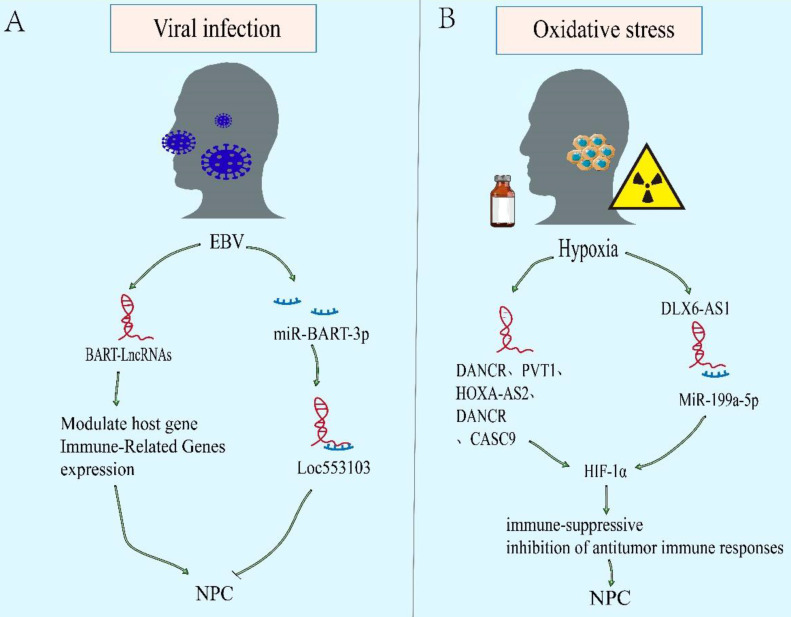
** LncRNAs as modulators of microenvironment associated with NPC. A.** EBV-encoded virus associated products can give NPC cells growth advantages in tumor microenvironment (TME).** B.** The oxidative stress microenvironment causing by many factors, such as drugs and radiotherapy, can activate the hypoxia signal pathway and affect the progress of NPC.

Epstein Barr Virus infection and oxidative stress caused by multiple factors bring unique microenvironment to NPC. The EBV-encoded virus associated products can give NPC cells growth advantages in tumor microenvironment (TME) 119. In addition, hypoxia microenvironment causing by many factors, such as drugs and radiotherapy, can activate the hypoxia signal pathway and affect the progress of NPC^120^, ^121^. Notably, the imbalance of lncRNAs is closely related to the EBV and hypoxia related microenvironment of NPC (Figure 4).

### Viral infection

EBV infection is an obvious feature of non-keratinizing NPC. The EBV-encoded viral products alter host cell signaling to facilitate tumor development and progression^122^, ^123^. Latent EBV exists only in tumor cell, mainly expressing EBV nuclear antigen 1 (EBNA1), EBV-encoded RNAs (EBERs), and elevated levels of BamHI-A rightward transcripts (BARTs) RNA^119^, ^124^. In addition, abnormal expression of lncRNAs is also found in EBV-infected. Evidences show that EBV can further regulate the downstream carcinogenic or tumor suppressor molecules via lncRNAs (Figure 4A). In NPC, EBV expresses few viral proteins but elevates levels of BARTs RNA, including viral microRNAs and lncRNAs^125^-^127^. And it also has long been suggested that EBV-infected NPC cells represents a unique TME. The lncRNA BART modulates host gene and immune-related genes expression, generates a cellular environment which supports EBV latency, and drives the oncogenic process^31^, ^128^. However, other than encoding oncogenes and promoting nasopharyngeal epithelium malignant transformation, some genes encoded by EBV can also inhibit the phenotype. However, there are few studies on the regulation of lncRNAs by EBV in NPC, and more evidence are needed to explain the mechanism.

### Oxidative stress

Most tumor cells are in a state of high metabolism and rapid proliferation. Besides, some factors, such as chemotherapy and radiotherapy, result in the continuous decline of oxygen content in the microenvironment. Eventually, a hypoxic microenvironment is often formed. However, tumor hypoxia is a significant factor leading to tumor treatment failure, recurrence and metastasis, due to the more aggressive ability of hypoxic tumor cells than normal oxygen cells^129^. Over the above process, hypoxia inducible factor-1 α (HIF-1 α) plays an important role in survival of hypoxic tumor cells, including NPC^130^, ^131^. Compared with normal nasopharyngeal epithelium, HIF-1 α is overexpressed and associates with TNM stage, lymph node metastasis, distant metastasis, and poor prognosis of NPC^132^. The downstream effects of an enhanced HIF-1α expression include (a) an activation of immune-suppressive effects (recruitment and stimulation of immune-suppressor cells [e.g., Treg, MDSC], and secretion of immune-suppressive TH2-type cytokines). And (b) inhibition of antitumor immune responses (including immune cell actions [e.g., NK, NKT, CD4+, CD8+], antigen-presenting cells [e.g., DC], and production of immune-stimulatory TH1-type cytokines)^133^. There is increasing evidence to support that lncRNAs may take part in hypoxia regulation related cancer progression including NPC (Figure 4B)^39^, ^134^, ^135^. The DANCR is initially discovered to be a carcinogen in hepatocellular carcinoma (HCC)^136^. Bioinformatics analysis suggested that DANCR is related to NPC metastasis and hypoxia phenotype. Mechanistically, DANCR interacted with the RNA-binding protein NF90/NF45 complex to stabilize HIF-1 α mRNA, leading to NPC Hypoxia tolerance and metastasis^65^. In addition, lncRNAs such as PVT1, HOXA-AS2 and DLX6-AS1 are also found to regulate HIF-1 α expression^39^, ^134^, ^135^.

### Diagnostic and therapeutic potentials of lncRNA in NPC

Up to now, MRI, CT and к ⁸ F-FDG-PET / CT are the most commonly used imaging methods for staging and curing NPC^137^. Yet, this kind of surveillance imaging is not enough to show early NPC. what's more, although EBV DNA testing can divide NPC patients into different risk subgroups, there is still lack of biomarkers with high specificity and sensitivity for NPC. Accordingly, emerging diagnostic and prognostic methods aim to increase accuracy in a more economical and non-invasive manner. In addition to the lack of reliable biomarkers, limited treatment options are also the causes of NPC related death. Although the survival rate of early NPC after radiotherapy and chemotherapy is high, the survival rate of patients with metastatic or recurrent NPC is still low. Therefore, new biomarkers and therapeutic targets are urgently needed to improve the diagnosis and treatment of NPC. Recently, the in-depth study of lncRNAs in NPC will provide new clinical utility for early diagnosis and treatment of NPC.

### LncRNAs are potential biomarkers for the diagnosis and treatment of NPC

LncRNAs are considered as a new biomarker of diseases. Many lncRNAs show species-specific and tissue-specific expression, which has potential applications in clinical oncology and become possible diagnostic biomarkers and predictors of therapeutic response.

As mentioned above, compared with normal nasal mucosa, there are a large number of unusual levels of lncRNAs in NPC tissues, which are helpful to distinguish NPC patients from normal people. The Genome sequencing showed that 306 lncRNAs are up-regulated, while 204 lncRNAs are down regulated^138^. Although only a few known lncRNAs have function annotation. These differentially expressed lncRNAs have potential to be new biomarkers of NPC. Like linc00312, the ROC curve showed that the level of linc00312 can distinguish NPC patients from non-tumor patients, with a sensitivity of 72.1% and a specificity of 87.7%, suggesting that linc00312 could be a potential biomarker for diagnosis of NPC^139^.

Circulating or secretory lncRNAs can also play important roles in clinical diagnosis^140^. Compared with solid tumor tissue sampling, liquid biopsy is an ideal non-invasive diagnostic method. Importantly, there may be multiple NPC related lncRNAs in body fluids (circulating lncRNAs). In the serum circulation of NPC patients, lncRNA MALAT1, APAF1-AS1 and AL359062 are markedly up-regulated and correlated with poor prognosis, indicating that they can be used as biomarkers for predicting poor outcome of NPC^141^. Representative NPC promoted lncRNAs can be detected in blood samples, and can be easily quantified by traditional qPCR^142^, which suggests a non-invasive method for the diagnosis of NPC by circulating lncRNAs detection.

Of course, various lncRNAs may also be potential prognostic markers for NPC. The lncRNA ANRIL is also an independent prognostic biomarker for local recurrence of NPC patients, which is higher in patients in stage III - IV than in stage I - II^87^. Not surprisingly, lncRNAs are involved in tumor metastasis may have good prognostic value. Higher linc01420 is apparently associated with distant metastasis and lower overall survival of NPC^143^.

LncRNAs play a vital role in the treatment of NPC. It indicates good anti-cancer effect by regulating their functions, which means that it may become a new target of NPC treatment. Like knockout of MINCR and PTPRG-AS1, which can enhance the sensitivity of NPC to X-ray^144^, ^145^. The MRVI1-AS1 can increase paclitaxel chemosensitivity of NPC by modulating the Hippo-TAZ signaling pathway^146^. The LINC00346 regulates the cisplatin resistance of NPC cells by inhibiting miR-342-5p, which could provide a potential target for chemotherapy resistance^147^. Although ARHGAP42 protein in metastatic NPC is obviously higher than that in primary NPC, the silence of its antisense lncRNA uc010rul resulted in decreased ARHGAP42 and significant inhibition of NPC migration and invasion^148^. Furthermore, potential role of lncRNA GAS5 polymorphisms rs2067079 and rs6790 as predictive biomarkers for chemoradiotherapy induced toxic reactions in NPC patients^149^.

### LncRNAs as promising NPC therapeutic targets

So far, the prognosis of advanced NPC still needs to be improved. Although PD-L1 has been used in the treatment of advanced NPC, the efficacy still needs further observation. Hence, the new goal is necessary to improve the prognosis of NPC. RNA-based therapeutics against cancer has gradually changed from concept to reality. The key role of lncRNAs in NPC makes it possible to become a new drug target for therapeutic interventions. These methods of targeting lncRNA mainly include antisense oligonucleotides (ASO) and RNA interference (RNAi), both of which show good anticancer activity by targeting lncRNAs.

Antisense oligonucleotides, a single strand DNA of 15-20 nucleotides, which can recognize specific sequences and degrade lncRNA by RNase-H^150^. They bind to RNA via standard Watson-Crick base pairing. After binding with target RNA, gene expression can be inhibited or changed by steric hindrance, splicing change, target degradation initiation or other events^151^. Currently, ASOs targeting different mRNAs have entered into clinical trials for several diseases including cancer^151^, emerging as a promising therapeutic approach for targeting lncRNAs^152^. Moreover, in order to achieve RNase H-mediated knockdown of target RNAs, chimeric ASOs referred to as 'GapmeRs' are used. GapmerR ASOs are RNA-DNA-RNA hybrids, which allows RNase H-mediated target RNA to extend and cleave in the center of DNA, while increasing target affinity and resistance to nuclease activity^153^. In NPC, knocking down of BART RNA is achieved by targeting it for GapmeR-mediated RNase H cleavage to disrupt its function, and successfully decreasing the level of BART, thus inhibiting immune evasion, progression, and metastasis of NPC^31^.

The siRNA is a double stranded RNA with 19-30 nucleotides. It can complementally bind to lncRNAs sequence through RNA mediated silencing complex (RISC) to degrade target lncRNAs^154^. Nevertheless, the preclinical study of targeting lncRNA with siRNAs / shRNA is very limited. And in the research of Li et al. the intracellular level of lncRNA‐ROR is degraded after transcription with siRNA ROR. As a result, it can inhibit the proliferation and migration of NPC and improve the chemical resistance^155^.

## Conclusions

With the deep research, lncRNAs are thought to be a new frontier for many diseases including malignant tumors. So that, A detailed understanding of their expression and mechanisms of action are critical in order to appreciate the importance of this unique class of molecules. Remarkably, the fact that lncRNAs are generally expressed in a cell- or tissue-specific manners make them to be exceptional therapeutic targets. Although many questions and challenges remain to be addressed, sequence-based nucleic acid therapeutics are evolving at a rapid pace. Identification and evaluation of a lncRNA target in the context of cancers may be translated into clinical scenarios in a reasonable time window.

## Figures and Tables

**Table 1:** Some dysregulated lncRNAs and their roles in the progression of NPC

lncRNAs	Roles in NPC	Biding partners	Action modes	Outcomes	Refs
HOXA-AS2	Oncogene	miR-519	miRNA sponge	↑proliferation↑metastasis	134
AFAP1-AS1	Oncogene	KAT2B protein	Histone modification	↑proliferation	37
PVT1	Oncogene	KAT2A protein	Cytoskeleton reorganization	↑proliferation	39
ANCR	Oncogene	EZH2 protein	Cytoskeleton reorganization	↑proliferation	38
CASC15	Oncogene	miR-101-3p	miRNA sponge	↑proliferation↑metastasis	156
ANRIL	Oncogene	miR-125a	miRNA sponge	↑proliferation↓Apoptosis	56
FAM225A	Oncogene	miR-590-3p miR-1275	miRNA sponge	↑proliferation ↑metastasis	21
ZNF667-AS1	Tumor suppressor	miR-1290	miRNA sponge	↓proliferation	157
MEG3	Tumor suppressor	miR‐21	miRNA sponge	↓autophagy ↓apoptosis	158
LINC01116	Oncogene	MYC mRNA	protein translation	↑proliferation↑metastasis	27
NPCCAT1	Oncogene	YY1mRNA	protein translation	↑proliferation ↑metastasis	58
THOR	Oncogene	YAP protein	protein localization	↑stemness	59
AWPPH	Oncogene	IGF2BP1 protein	Protein stabilization	↑proliferation ↓Apoptosis	159
DANCR	Oncogene	RBM3 protein	Cytoskeleton reorganization	↑proliferation	66
LINC01133	Oncogene	YBX1 protein	sequestration of proteins	↑proliferation ↑metastasis	69
LINC00669	Oncogene	SOCS1	sequestration of proteins	↑proliferation↑metastasis	61
LET	Tumor suppressor	ERK1/2	Protein phosphorylation	↓proliferation ↓metastasis	75
MACC1-AS1	Oncogene	miR-145	miRNA sponge	↑stemness	102
FOXD1-AS1	Oncogene	FOXD1	protein translation	↑glycolysis	88
TINCR	Oncogene	ACLY	Avoid ubiquitination degradation	↑Acetyl-Coa Metabolism	93
HCG18	Oncogene	miR-140	miRNA sponge	↑proliferation ↑metastasis	55
EPB41L4A-AS2	Oncogene	YBX1 protein	miRNA sponge	↑metastasis	68
RP11-624L4.1	Oncogene	CDK4, CDK6, and Cyclin D1	sequestration of protein	↑proliferation	73
MALAT1	Oncogene	miR-124	miRNA sponge	↑proliferation ↑metastasis	45
HOTAIR	Oncogene	VEGFA mRNA	transcription	↑Angiogenesis	115

## References

[1] Wang Y, Song Q, Huang X, Chen Z, Zhang F, Wang K (2019). Long noncoding RNA GAS5 promotes apoptosis in primary nucleus pulposus cells derived from the human intervertebral disc via Bcl2 downregulation and caspase3 upregulation. Molecular medicine reports.

[2] Bray F, Ferlay J, Soerjomataram I, Siegel RL, Torre LA, Jemal A (2018). Global cancer statistics 2018: GLOBOCAN estimates of incidence and mortality worldwide for 36 cancers in 185 countries. CA: a cancer journal for clinicians.

[3] Yao JJ, Jin YN, Liu ZG, Liu QD, Pei XF, Zhou HL (2019). Do all patients with advanced N-stage nasopharyngeal carcinoma benefit from the addition of induction chemotherapy to concurrent chemoradiotherapy?. Therapeutic advances in medical oncology.

[4] Li Y, Ou X, Shen C, Xu T, Li W, Hu C (2018). Patterns of local failures and suggestions for reduction of clinical target volume for nasopharyngeal carcinoma patients without cervical lymph node metastasis. OncoTargets and therapy.

[5] Colevas AD, Yom SS, Pfister DG, Spencer S, Adelstein D, Adkins D (2018). NCCN Guidelines Insights: Head and Neck Cancers, Version 1.2018. Journal of the National Comprehensive Cancer Network: JNCCN.

[6] The ENCODE (ENCyclopedia Of DNA Elements) Project Science (New York, NY). 2004; 306: 636-40.

[7] Iyer MK, Niknafs YS, Malik R, Singhal U, Sahu A, Hosono Y (2015). The landscape of long noncoding RNAs in the human transcriptome. Nature genetics.

[8] Sarfi M, Abbastabar M, Khalili E (2019). Long noncoding RNAs biomarker-based cancer assessment. Journal of cellular physiology.

[9] Bach DH, Lee SK (2018). Long noncoding RNAs in cancer cells. Cancer Lett.

[10] Bijnsdorp IV, van Royen ME, Verhaegh GW, Martens-Uzunova ES (2017). The Non-Coding Transcriptome of Prostate Cancer: Implications for Clinical Practice. Molecular diagnosis & therapy.

[11] Jiang MC, Ni JJ, Cui WY, Wang BY, Zhuo W (2019). Emerging roles of lncRNA in cancer and therapeutic opportunities. American journal of cancer research.

[12] Huang JZ, Chen M, Chen D, Gao XC, Zhu S, Huang H (2017). A Peptide Encoded by a Putative lncRNA HOXB-AS3 Suppresses Colon Cancer Growth. Molecular cell.

[13] Lin A, Li C, Xing Z, Hu Q, Liang K, Han L (2016). The LINK-A lncRNA activates normoxic HIF1α signalling in triple-negative breast cancer. Nature cell biology.

[14] Xiao ZD, Han L, Lee H, Zhuang L, Zhang Y, Baddour J (2017). Energy stress-induced lncRNA FILNC1 represses c-Myc-mediated energy metabolism and inhibits renal tumor development. Nature communications.

[15] Slaby O, Laga R, Sedlacek O (2017). Therapeutic targeting of non-coding RNAs in cancer. Biochemical Journal.

[16] Jones PA (2002). DNA methylation and cancer. Oncogene.

[17] Jones PA, Takai D (2001). The role of DNA methylation in mammalian epigenetics. Science (New York, NY).

[18] Zhu WG, Dai Z, Ding H, Srinivasan K, Hall J, Duan W (2001). Increased expression of unmethylated CDKN2D by 5-aza-2'-deoxycytidine in human lung cancer cells. Oncogene.

[19] Sun Q, Liu H, Li L, Zhang S, Liu K, Liu Y (2015). Long noncoding RNA-LET, which is repressed by EZH2, inhibits cell proliferation and induces apoptosis of nasopharyngeal carcinoma cell. Medical oncology (Northwood, London, England).

[20] Sun W, Chen L, Tang J, Zhang C, Wen Y, Wen W (2020). Targeting EZH2 depletes LMP1-induced activated regulatory T cells enhancing antitumor immunity in nasopharyngeal carcinoma. Journal of cancer research and therapeutics.

[21] Zheng ZQ, Li ZX, Zhou GQ, Lin L, Zhang LL, Lv JW (2019). Long Noncoding RNA FAM225A Promotes Nasopharyngeal Carcinoma Tumorigenesis and Metastasis by Acting as ceRNA to Sponge miR-590-3p/miR-1275 and Upregulate ITGB3. Cancer Res.

[22] Wu JH, Tang JM, Li J, Li XW (2018). Upregulation of SOX2-activated lncRNA ANRIL promotes nasopharyngeal carcinoma cell growth. Scientific reports.

[23] He SW, Xu C, Li YQ, Li YQ, Zhao Y, Zhang PP (2020). AR-induced long non-coding RNA LINC01503 facilitates proliferation and metastasis via the SFPQ-FOSL1 axis in nasopharyngeal carcinoma. Oncogene.

[24] Wei F, Jing YZ, He Y, Tang YY, Yang LT, Wu YF (2019). Cloning and characterization of the putative AFAP1-AS1 promoter region. Journal of Cancer.

[25] Pelengaris S, Khan M, Evan G (2002). c-MYC: more than just a matter of life and death. Nat Rev Cancer.

[26] Kenneth NS, White RJ (2009). Regulation by c-Myc of ncRNA expression. Current opinion in genetics & development.

[27] Xing H, Sun H, Du W (2020). LINC01116 accelerates nasopharyngeal carcinoma progression based on its enhancement on MYC transcription activity. Cancer medicine.

[28] Hua WF, Zhong Q, Xia TL, Chen Q, Zhang MY, Zhou AJ (2016). RBM24 suppresses cancer progression by upregulating miR-25 to target MALAT1 in nasopharyngeal carcinoma. Cell death & disease.

[29] Leucci E, Patella F, Waage J, Holmstrøm K, Lindow M, Porse B (2013). microRNA-9 targets the long non-coding RNA MALAT1 for degradation in the nucleus. Scientific reports.

[30] Edwards RH, Marquitz AR, Raab-Traub N (2008). Epstein-Barr virus BART microRNAs are produced from a large intron prior to splicing. Journal of virology.

[31] Verhoeven RJA, Tong S, Mok BW, Liu J, He S, Zong J (2019). Epstein-Barr Virus BART Long Non-coding RNAs Function as Epigenetic Modulators in Nasopharyngeal Carcinoma. Frontiers in oncology.

[32] Huarte M (2015). The emerging role of lncRNAs in cancer. Nat Med.

[33] Noh JH, Kim KM, McClusky WG, Abdelmohsen K, Gorospe M (2018). Cytoplasmic functions of long noncoding RNAs. WIRES RNA.

[34] Yao RW, Wang Y, Chen LL (2019). Cellular functions of long noncoding RNAs. Nature cell biology.

[35] Wei JW, Huang K, Yang C, Kang CS (2017). Non-coding RNAs as regulators in epigenetics (Review). Oncology reports.

[36] Kondo Y, Shinjo K, Katsushima K (2017). Long non-coding RNAs as an epigenetic regulator in human cancers. Cancer science.

[37] Fang M, Zhang M, Wang Y, Wei F, Wu J, Mou X (2020). Long Noncoding RNA AFAP1-AS1 Is a Critical Regulator of Nasopharyngeal Carcinoma Tumorigenicity. Frontiers in oncology.

[38] Ma X, Zhou J, Liu J, Wu G, Yu Y, Zhu H (2018). LncRNA ANCR promotes proliferation and radiation resistance of nasopharyngeal carcinoma by inhibiting PTEN expression. OncoTargets and therapy.

[39] Wang Y, Chen W, Lian J, Zhang H, Yu B, Zhang M (2020). The lncRNA PVT1 regulates nasopharyngeal carcinoma cell proliferation via activating the KAT2A acetyltransferase and stabilizing HIF-1伪. Cell death and differentiation.

[40] Bird A (2002). DNA methylation patterns and epigenetic memory. Genes & development.

[41] Ng A, Tang JP, Goh CH, Hui KM (2003). Regulation of the H19 imprinting gene expression in human nasopharyngeal carcinoma by methylation. Int J Cancer.

[42] Xu Y, Huang X, Ye W, Zhang Y, Li C, Bai P (2020). Comprehensive analysis of key genes associated with ceRNA networks in nasopharyngeal carcinoma based on bioinformatics analysis. Cancer cell international.

[43] Lan X, Liu X (2019). LncRNA SNHG1 functions as a ceRNA to antagonize the effect of miR-145a-5p on the down-regulation of NUAK1 in nasopharyngeal carcinoma cell. Journal of cellular and molecular medicine.

[44] Chen W, Du M, Hu X, Ma H, Zhang E, Wang T (2020). Long noncoding RNA cytoskeleton regulator RNA promotes cell invasion and metastasis by titrating miR-613 to regulate ANXA2 in nasopharyngeal carcinoma. Cancer medicine.

[45] Shi B, Wang Y, Yin F (2017). MALAT1/miR-124/Capn4 axis regulates proliferation, invasion and EMT in nasopharyngeal carcinoma cells. Cancer biology & therapy.

[46] Lu Y, Li T, Wei G, Liu L, Chen Q, Xu L (2016). The long non-coding RNA NEAT1 regulates epithelial to mesenchymal transition and radioresistance in through miR-204/ZEB1 axis in nasopharyngeal carcinoma. Tumour biology: the journal of the International Society for Oncodevelopmental Biology and Medicine.

[47] Chan JJ, Tay Y (2018). Noncoding RNA:RNA Regulatory Networks in Cancer. International journal of molecular sciences.

[48] Ji Y, Wang M, Li X, Cui F (2019). The Long Noncoding RNA NEAT1 Targets miR-34a-5p and Drives Nasopharyngeal Carcinoma Progression via Wnt/beta-Catenin Signaling. Yonsei medical journal.

[49] Zhang W, Zhang Y, Xi S (2019). Upregulation of lncRNA HAGLROS enhances the development of nasopharyngeal carcinoma via modulating miR-100/ATG14 axis-mediated PI3K/AKT/mTOR signals. Artificial cells, nanomedicine, and biotechnology.

[50] Xie T, Pi G, Yang B, Ren H, Yu J, Ren Q (2019). Long non-coding RNA 520 is a negative prognostic biomarker and exhibits pro-oncogenic function in nasopharyngeal carcinoma carcinogenesis through regulation of miR-26b-3p/USP39 axis. Gene.

[51] Zhou M, Dong Z, Hu S, Xiao M (2021). LINC01433 targets miR-506-3p to promote the biological progress of nasopharyngeal carcinoma cells. European archives of oto-rhino-laryngology: official journal of the European Federation of Oto-Rhino-Laryngological Societies (EUFOS): affiliated with the German Society for Oto-Rhino-Laryngology - Head and Neck Surgery.

[52] Zhang S, Li P, Zhao L, Xu L (2018). LINC00210 as a miR-328-5p sponge promotes nasopharyngeal carcinoma tumorigenesis by activating NOTCH3 pathway. Bioscience reports.

[53] Liu F, Tai Y, Ma J (2018). LncRNA NEAT1/let-7a-5p axis regulates the cisplatin resistance in nasopharyngeal carcinoma by targeting Rsf-1 and modulating the Ras-MAPK pathway. Cancer biology & therapy.

[54] Cheng N, Guo Y (2017). Long noncoding RNA NEAT1 promotes nasopharyngeal carcinoma progression through regulation of miR-124/NF-κB pathway. OncoTargets and therapy.

[55] Li L, Ma TT, Ma YH, Jiang YF (2019). LncRNA HCG18 contributes to nasopharyngeal carcinoma development by modulating miR-140/CCND1 and Hedgehog signaling pathway. European review for medical and pharmacological sciences.

[56] Hu X, Jiang H, Jiang X (2017). Downregulation of lncRNA ANRIL inhibits proliferation, induces apoptosis, and enhances radiosensitivity in nasopharyngeal carcinoma cells through regulating miR-125a. Cancer biology & therapy.

[57] Ingolia NT, Lareau LF, Weissman JS (2011). Ribosome profiling of mouse embryonic stem cells reveals the complexity and dynamics of mammalian proteomes. Cell.

[58] Su H, Liu L, Zhang Y, Wang J, Zhao Y (2019). Long noncoding RNA NPCCAT1 promotes nasopharyngeal carcinoma progression via upregulating YY1. Biochimie.

[59] Gao L, Cheng XL, Cao H (2018). LncRNA THOR attenuates cisplatin sensitivity of nasopharyngeal carcinoma cells via enhancing cells stemness. Biochimie.

[60] Liu YP, Tan YN, Wang ZL, Zeng L, Lu ZX, Li LL (2008). Phosphorylation and nuclear translocation of STAT3 regulated by the Epstein-Barr virus latent membrane protein 1 in nasopharyngeal carcinoma. International journal of molecular medicine.

[61] Qing X, Tan GL, Liu HW, Li W, Ai JG, Xiong SS (2020). LINC00669 insulates the JAK/STAT suppressor SOCS1 to promote nasopharyngeal cancer cell proliferation and invasion. Journal of experimental & clinical cancer research: CR.

[62] Zhang X, Yang J, Bian Z, Shi D, Cao Z (2019). Long noncoding RNA DANCR promotes nasopharyngeal carcinoma progression by interacting with STAT3, enhancing IL-6/JAK1/STAT3 signaling. Biomedicine & pharmacotherapy = Biomedecine & pharmacotherapie.

[63] Maiques-Diaz A, Somervaille TC (2016). LSD1: biologic roles and therapeutic targeting. Epigenomics.

[64] Schmitz SU, Grote P, Herrmann BG (2016). Mechanisms of long noncoding RNA function in development and disease. Cellular and molecular life sciences: CMLS.

[65] Wen X, Liu X, Mao YP, Yang XJ, Wang YQ, Zhang PP (2018). Long non-coding RNA DANCR stabilizes HIF-1α and promotes metastasis by interacting with NF90/NF45 complex in nasopharyngeal carcinoma. Theranostics.

[66] Li Q, Jiang Y, Zhong G, Lu Y, Song T, Zhang Y (2020). Long Noncoding RNA DANCR Regulates Cell Proliferation by Stabilizing SOX2 mRNA in Nasopharyngeal Carcinoma. The American journal of pathology.

[67] Evdokimova V, Tognon C, Ng T, Ruzanov P, Melnyk N, Fink D (2009). Translational activation of snail1 and other developmentally regulated transcription factors by YB-1 promotes an epithelial-mesenchymal transition. Cancer Cell.

[68] Du M, Hu X, Jiang X, Yin L, Chen J, Wen J (2021). LncRNA EPB41L4A-AS2 represses Nasopharyngeal Carcinoma Metastasis by binding to YBX1 in the Nucleus and Sponging MiR-107 in the Cytoplasm. International journal of biological sciences.

[69] Zhang W, Du M, Wang T, Chen W, Wu J, Li Q (2019). Long non-coding RNA LINC01133 mediates nasopharyngeal carcinoma tumorigenesis by binding to YBX1. American journal of cancer research.

[70] Gutschner T, Diederichs S (2012). The hallmarks of cancer: a long non-coding RNA point of view. RNA biology.

[71] Sherr CJ, Beach D, Shapiro GI (2016). Targeting CDK4 and CDK6: From Discovery to Therapy. Cancer discovery.

[72] Sherr CJ, Roberts JM (2004). Living with or without cyclins and cyclin-dependent kinases. Genes & development.

[73] Zhou L, Liu R, Liang X, Zhang S, Bi W, Yang M (2020). lncRNA RP11-624L4.1 Is Associated with Unfavorable Prognosis and Promotes Proliferation via the CDK4/6-Cyclin D1-Rb-E2F1 Pathway in NPC. Molecular therapy Nucleic acids.

[74] Meloche S, Pouyssegur J (2007). The ERK1/2 mitogen-activated protein kinase pathway as a master regulator of the G1- to S-phase transition. Oncogene.

[75] Chen L, Sun L, Dong L, Cui P, Xia Z, Li C (2017). The role of long noncoding RNA-LET in cell proliferation and invasion of nasopharyngeal carcinoma and its mechanism. OncoTargets and therapy.

[76] Zhang Y, Wang X (2020). Targeting the Wnt/β-catenin signaling pathway in cancer. Journal of hematology & oncology.

[77] Chen X, Li J, Li CL, Lu X (2018). Long non-coding RNA ZFAS1 promotes nasopharyngeal carcinoma through activation of Wnt/β-catenin pathway. European review for medical and pharmacological sciences.

[78] Cheng Y (2019). FEZF1-AS1 is a key regulator of cell cycle, epithelial-mesenchymal transition and Wnt/β-catenin signaling in nasopharyngeal carcinoma cells. Bioscience reports.

[79] Jiang X, Liu W (2017). Long Noncoding RNA Highly Upregulated in Liver Cancer Activates p53-p21 Pathway and Promotes Nasopharyngeal Carcinoma Cell Growth. DNA and cell biology.

[80] Warburg O (1956). On the origin of cancer cells. Science.

[81] Vander Heiden MG, Cantley LC, Thompson CB (2009). Understanding the Warburg effect: the metabolic requirements of cell proliferation. Science.

[82] DeBerardinis RJ, Mancuso A, Daikhin E, Nissim I, Yudkoff M, Wehrli S (2007). Beyond aerobic glycolysis: transformed cells can engage in glutamine metabolism that exceeds the requirement for protein and nucleotide synthesis. Proceedings of the National Academy of Sciences of the United States of America.

[83] Sun H, Huang Z, Sheng W, Xu MD (2018). Emerging roles of long non-coding RNAs in tumor metabolism. J Hematol Oncol.

[84] Fan C, Tang Y, Wang J, Xiong F, Guo C, Wang Y (2017). Role of long non-coding RNAs in glucose metabolism in cancer. Mol Cancer.

[85] Balon TW (2012). SGLT and GLUT: are they teammates? Focus on "Mouse SGLT3a generates proton-activated currents but does not transport sugar". American journal of physiology Cell physiology.

[86] Furuta E, Okuda H, Kobayashi A, Watabe K (2010). Metabolic genes in cancer: their roles in tumor progression and clinical implications. Biochimica et biophysica acta.

[87] Zou ZW, Ma C, Medoro L, Chen L, Wang B, Gupta R (2016). LncRNA ANRIL is up-regulated in nasopharyngeal carcinoma and promotes the cancer progression via increasing proliferation, reprograming cell glucose metabolism and inducing side-population stem-like cancer cells. Oncotarget.

[88] Wang Z, Cheng Y, Zhu Y, Hu X, Jin Y, Gong L (2020). Long non-coding RNA FOXD1-AS1 promotes the progression and glycolysis of nasopharyngeal carcinoma by sustaining FOXD1 expression. American journal of cancer research.

[89] Robey RB, Weisz J, Kuemmerle NB, Salzberg AC, Berg A, Brown DG (2015). Metabolic reprogramming and dysregulated metabolism: cause, consequence and/or enabler of environmental carcinogenesis?. Carcinogenesis.

[90] Menendez JA, Lupu R (2007). Fatty acid synthase and the lipogenic phenotype in cancer pathogenesis. Nature reviews Cancer.

[91] Rysman E, Brusselmans K, Scheys K, Timmermans L, Derua R, Munck S (2010). De novo lipogenesis protects cancer cells from free radicals and chemotherapeutics by promoting membrane lipid saturation. Cancer Res.

[92] Metallo CM, Gameiro PA, Bell EL, Mattaini KR, Yang J, Hiller K (2011). Reductive glutamine metabolism by IDH1 mediates lipogenesis under hypoxia. Nature.

[93] Zheng ZQ, Li ZX, Guan JL, Liu X, Li JY, Chen Y (2020). Long Noncoding RNA TINCR-Mediated Regulation of Acetyl-CoA Metabolism Promotes Nasopharyngeal Carcinoma Progression and Chemoresistance. Cancer research.

[94] Reya T, Morrison SJ, Clarke MF, Weissman IL (2001). Stem cells, cancer, and cancer stem cells. Nature.

[95] Ishiguro T, Ohata H, Sato A, Yamawaki K, Enomoto T, Okamoto K (2017). Tumor-derived spheroids: Relevance to cancer stem cells and clinical applications. Cancer science.

[96] Zhang W, Sui Y, Ni J, Yang T (2016). Insights into the Nanog gene: A propeller for stemness in primitive stem cells. International journal of biological sciences.

[97] Castro-Oropeza R, Melendez-Zajgla J, Maldonado V, Vazquez-Santillan K (2018). The emerging role of lncRNAs in the regulation of cancer stem cells. Cellular oncology (Dordrecht).

[98] Hu S, Shan G (2016). LncRNAs in Stem Cells. Stem cells international.

[99] Hadjimichael C, Chanoumidou K, Papadopoulou N, Arampatzi P, Papamatheakis J, Kretsovali A (2015). Common stemness regulators of embryonic and cancer stem cells. World J Stem Cells.

[100] Najafi M, Mortezaee K, Majidpoor J (2019). Cancer stem cell (CSC) resistance drivers. Life sciences.

[101] Meng Cui, Yu Chang, Qi-Gen Fang (2018). Non-Coding RNA Pvt1 Promotes Cancer Stem Cell-Like Traits in Nasopharyngeal Cancer via Inhibiting miR-1207. Pathol Oncol Res.

[102] Chen S, Luo X, Wu W, Li Y, Yu H, Wang Y (2020). The long non-coding RNA MACC1-AS1 promotes nasopharyngeal carcinoma cell stemness via suppressing miR-145-mediated inhibition on SMAD2/MACC1-AS1 axis. Biomedicine & pharmacotherapy = Biomedecine & pharmacotherapie.

[103] Lee JE, Park HS, Lee D, Yoo G, Kim T, Jeon H (2016). Hippo pathway effector YAP inhibition restores the sensitivity of EGFR-TKI in lung adenocarcinoma having primary or acquired EGFR-TKI resistance. Biochemical and biophysical research communications.

[104] Shah SR, David JM, Tippens ND, Mohyeldin A, Martinez-Gutierrez JC, Ganaha S (2017). Brachyury-YAP Regulatory Axis Drives Stemness and Growth in Cancer. Cell reports.

[105] Kim T, Yang SJ, Hwang D, Song J, Kim M, Kyum Kim S (2015). A basal-like breast cancer-specific role for SRF-IL6 in YAP-induced cancer stemness. Nature communications.

[106] Masuda T, Hayashi N, Iguchi T, Ito S, Eguchi H, Mimori K (2016). Clinical and biological significance of circulating tumor cells in cancer. Molecular oncology.

[107] Pastushenko I, Blanpain C (2019). EMT Transition States during Tumor Progression and Metastasis. Trends in cell biology.

[108] Wang X, Jin Q, Wang X, Chen W, Cai Z (2019). LncRNA ZFAS1 promotes proliferation and migration and inhibits apoptosis in nasopharyngeal carcinoma via the PI3K/AKT pathway in vitro. Cancer biomarkers: section A of Disease markers.

[109] Meng XJ, Wang JC, Wang AQ, Xia WQ (2020). Downregulation of lncRNA CCHE1 inhibits cell proliferation, migration and invasion by suppressing MEK/ERK/c-MYC pathway in nasopharyngeal carcinoma. European review for medical and pharmacological sciences.

[110] Fan C, Tang Y, Wang J, Wang Y, Xiong F, Zhang S (2019). Long non-coding RNA LOC284454 promotes migration and invasion of nasopharyngeal carcinoma via modulating the Rho/Rac signaling pathway. Carcinogenesis.

[111] Zhang W, Guo Q, Liu G, Zheng F, Chen J, Huang D (2019). NKILA represses nasopharyngeal carcinoma carcinogenesis and metastasis by NF-κB pathway inhibition. PLoS genetics.

[112] Folkman J (2006). Angiogenesis. Annual review of medicine.

[113] Hanahan D, Weinberg RA (2000). The hallmarks of cancer. Cell.

[114] Ferrara N (2002). VEGF and the quest for tumour angiogenesis factors. Nat Rev Cancer.

[115] Fu WM, Lu YF, Hu BG, Liang WC, Zhu X, Yang HD (2016). Long noncoding RNA Hotair mediated angiogenesis in nasopharyngeal carcinoma by direct and indirect signaling pathways. Oncotarget.

[116] Ferrara N, Gerber HP, LeCouter J (2003). The biology of VEGF and its receptors. Nat Med.

[117] Chen S, Lv L, Zhan Z, Wang X, You Z, Luo X (2019). Silencing of long noncoding RNA SRRM2-AS exerts suppressive effects on angiogenesis in nasopharyngeal carcinoma via activating MYLK-mediated cGMP-PKG signaling pathway. Journal of cellular physiology.

[118] Folkman J Angiogenesis. 2006; 57: 1-18.

[119] Elgui de Oliveira D, Müller-Coan BG, Pagano JS (2016). Viral Carcinogenesis Beyond Malignant Transformation: EBV in the Progression of Human Cancers. Trends in microbiology.

[120] Jarosz-Biej M, Smolarczyk R, Cicho T, Ku?ach N (2019). Tumor Microenvironment as A "Game Changer" in Cancer Radiotherapy. International journal of molecular sciences.

[121] Jing X, Yang F, Shao C, Wei K, Xie M, Shen H (2019). Role of hypoxia in cancer therapy by regulating the tumor microenvironment. Molecular cancer.

[122] Huang SCM, Tsao SW, Tsang CM (2018). Interplay of Viral Infection, Host Cell Factors and Tumor Microenvironment in the Pathogenesis of Nasopharyngeal Carcinoma. Cancers (Basel).

[123] Yang Z, Wang J, Zhang Z, Tang F (2019). Epstein-Barr Virus-Encoded Products Promote Circulating Tumor Cell Generation: A Novel Mechanism of Nasopharyngeal Carcinoma Metastasis. OncoTargets and therapy.

[124] Young LS, Dawson CW (2014). Epstein-Barr virus and nasopharyngeal carcinoma. Chinese journal of cancer.

[125] Chen HL, Lung MM, Sham JS, Choy DT, Griffin BE, Ng MH (1992). Transcription of BamHI-A region of the EBV genome in NPC tissues and B cells. Virology.

[126] Hitt MM, Allday MJ, Hara T, Karran L, Jones MD, Busson P (1989). EBV gene expression in an NPC-related tumour. The EMBO journal.

[127] Gilligan K, Sato H, Rajadurai P, Busson P, Young L, Rickinson A (1990). Novel transcription from the Epstein-Barr virus terminal EcoRI fragment, DIJhet, in a nasopharyngeal carcinoma. Journal of virology.

[128] Verhoeven RJ, Tong S, Zhang G, Zong J, Chen Y, Jin DY (2016). NF-κB Signaling Regulates Expression of Epstein-Barr Virus BART MicroRNAs and Long Noncoding RNAs in Nasopharyngeal Carcinoma. Journal of virology.

[129] Warfel N, Sainz AG, Song JH, Kraft A (2016). PIM Kinase Inhibitors Kill Hypoxic Tumor Cells by Reducing Nrf2 Signaling and Increasing Reactive Oxygen Species. Mol Cancer Ther.

[130] Wan XB, Fan XJ, Huang PY, Dong D, Zhang Y, Chen MY (2012). Aurora-A activation, correlated with hypoxia-inducible factor-1alpha, promotes radiochemoresistance and predicts poor outcome for nasopharyngeal carcinoma. Cancer science.

[131] Shou Z, Lin L, Liang J, Li JL, Chen HY (2012). Expression and prognosis of FOXO3a and HIF-1alpha in nasopharyngeal carcinoma. Journal of cancer research and clinical oncology.

[132] Wu Shihai, Xu Gang, Chen Yuhan (2014). Expression of HIF-1 alpha and CAIX in nasopharyngeal carcinoma and their correlation with patients' prognosis. Medical oncology (Northwood, London, England).

[133] Vaupel P, Multhoff G (2018). Hypoxia-/HIF-1α-Driven Factors of the Tumor Microenvironment Impeding Antitumor Immune Responses and Promoting Malignant Progression. Advances in experimental medicine and biology.

[134] Wang S, You H, Yu S (2020). Long non-coding RNA HOXA-AS2 promotes the expression levels of hypoxia-inducible factor-1α and programmed death-ligand 1, and regulates nasopharyngeal carcinoma progression via miR-519. Oncology letters.

[135] Yang B, Jia L, Ren H, Jin C, Ren Q, Zhang H (2020). LncRNA DLX6-AS1 increases the expression of HIF-1α and promotes the malignant phenotypes of nasopharyngeal carcinoma cells via targeting MiR-199a-5p. Molecular genetics & genomic medicine.

[136] Long noncoding RNA DANCR increases stemness features of hepatocellular carcinoma by derepression of CTNNB1 Hepatology. 2016; 63.

[137] Chen YP, Chan ATC, Le QT, Blanchard P, Sun Y, Ma J (2019). Nasopharyngeal carcinoma. Lancet (London, England).

[138] Li XX, Liang XJ, Zhou LY, Liu RJ, Bi W, Zhang S (2018). Analysis of Differential Expressions of Long Non-coding RNAs in Nasopharyngeal Carcinoma Using Next-generation Deep Sequencing. Journal of Cancer.

[139] Zhang W, Huang C, Gong Z, Zhao Y, Tang K, Li X (2013). Expression of LINC00312, a long intergenic non-coding RNA, is negatively correlated with tumor size but positively correlated with lymph node metastasis in nasopharyngeal carcinoma. Journal of molecular histology.

[140] Xie Y, Dang W, Zhang S, Yue W, Yang L, Zhai X (2019). The role of exosomal noncoding RNAs in cancer. Mol Cancer.

[141] He B, Zeng J, Chao W, Chen X, Huang Y, Deng K (2017). Serum long non-coding RNAs MALAT1, AFAP1-AS1 and AL359062 as diagnostic and prognostic biomarkers for nasopharyngeal carcinoma. Oncotarget.

[142] Vaidyanathan R, Soon RH, Zhang P, Jiang K, Lim CT (2018). Cancer diagnosis: from tumor to liquid biopsy and beyond. Lab on a chip.

[143] Yang L, Tang Y, He Y, Wang Y, Lian Y, Xiong F (2017). High Expression of LINC01420 indicates an unfavorable prognosis and modulates cell migration and invasion in nasopharyngeal carcinoma. Journal of Cancer.

[144] Yi L, Ouyang L, Wang S, Li SS, Yang XM (2019). Long noncoding RNA PTPRG-AS1 acts as a microRNA-194-3p sponge to regulate radiosensitivity and metastasis of nasopharyngeal carcinoma cells via PRC1. Journal of cellular physiology.

[145] Zhong Q, Chen Y, Chen Z (2020). LncRNA MINCR regulates irradiation resistance in nasopharyngeal carcinoma cells via the microRNA-223/ZEB1 axis. Cell cycle (Georgetown, Tex).

[146] Zhu Y, He D, Bo H, Liu Z, Xiao M, Xiang L (2019). The MRVI1-AS1/ATF3 signaling loop sensitizes nasopharyngeal cancer cells to paclitaxel by regulating the Hippo-TAZ pathway. Oncogene.

[147] Cui Z, Pu T, Zhang Y, Wang J, Zhao Y (2020). Long non-coding RNA LINC00346 contributes to cisplatin resistance in nasopharyngeal carcinoma by repressing miR-342-5p. Open biology.

[148] Hu Q, Lin X, Ding L, Zeng Y, Pang D, Ouyang N (2018). ARHGAP42 promotes cell migration and invasion involving PI3K/Akt signaling pathway in nasopharyngeal carcinoma. Cancer medicine.

[149] Guo Z, Wang Y, Zhao Y, Jin Y, An L, Wu B (2017). Genetic polymorphisms of long non-coding RNA GAS5 predict platinum-based concurrent chemoradiotherapy response in nasopharyngeal carcinoma patients. Oncotarget.

[150] Allerson CR, Sioufi N, Jarres R, Prakash TP, Naik N, Berdeja A (2005). Fully 2'-modified oligonucleotide duplexes with improved in vitro potency and stability compared to unmodified small interfering RNA. Journal of medicinal chemistry.

[151] Bennett CF, Baker BF, Pham N, Swayze E, Geary RS (2017). Pharmacology of Antisense Drugs. Annual review of pharmacology and toxicology.

[152] Arun G, Diermeier S, Akerman M, Chang KC, Wilkinson JE, Hearn S (2016). Differentiation of mammary tumors and reduction in metastasis upon Malat1 lncRNA loss. Genes & development.

[153] Yu RZ, Grundy JS, Geary RS (2013). Clinical pharmacokinetics of second generation antisense oligonucleotides. Expert opinion on drug metabolism & toxicology.

[154] Hannon GJ, Rossi JJ (2004). Unlocking the potential of the human genome with RNA interference. Nature.

[155] Li L, Gu M, You B, Shi S, Shan Y, Bao L (2016). Long non-coding RNA ROR promotes proliferation, migration and chemoresistance of nasopharyngeal carcinoma. Cancer science.

[156] Xue MY, Cao HX (2019). Long non-coding RNA CASC15 promotes nasopharyngeal carcinoma cell proliferation and metastasis by downregulating miR-101-3p. European review for medical and pharmacological sciences.

[157] Chen X, Huang Y, Shi D, Nie C, Luo Y, Guo L (2020). LncRNA ZNF667-AS1 Promotes ABLIM1 Expression by Adsorbing micro RNA-1290 to Suppress Nasopharyngeal Carcinoma Cell Progression. OncoTargets and therapy.

[158] Lin L, Liu X, Lv B (2021). Long non-coding RNA MEG3 promotes autophagy and apoptosis of nasopharyngeal carcinoma cells via PTEN up-regulation by binding to microRNA-21. Journal of cellular and molecular medicine.

[159] Guo D, Liu F, Zhang L, Bian N, Liu L, Kong L (2020). Long non-coding RNA AWPPH enhances malignant phenotypes in nasopharyngeal carcinoma via silencing PTEN through interacting with LSD1 and EZH2. Biochemistry and cell biology = Biochimie et biologie cellulaire.

